# Low urinary selenium concentration is associated with nonthyroidal illness syndrome in hospitalized patients with COVID-19

**DOI:** 10.20945/2359-4292-2024-0113

**Published:** 2025-05-31

**Authors:** Sara Moreira Anunciação, Renata de Oliveira Campos, Fabyan Esberard de Lima Beltrão, Déborah Araújo Morais, Wellington Tavares de Sousa Júnior, Fernando Barbosa Júnior, Jéssica Fernanda Cassemiro, Pedro Resende Ferreira Rende, Fabio Hecht, Helton Estrela Ramos

**Affiliations:** 1 Departamento de Biorregulação, Instituto de Saúde e Ciências, Universidade Federal da Bahia, Salvador, BA, Brasil; 2 Programa de Pós-graduação em Processos Interativos de Órgãos e Sistemas, Instituto de Ciências e Saúde, Universidade Federal da Bahia, Salvador, BA, Brasil; 3 Centro de Ciências e Saúde, Recôncavo da Universidade Federal da Bahia, Santo Antonio de Jesus, BA, Brasil; 4 Hospital Universitário Lauro Wanderley, Universidade Federal da Paraíba, João Pessoa, PB, Brasil; 5 Programa de Pós-graduação em Ciências da Nutrição, Departamento de Nutrição, Centro de Ciências da Saúde, Universidade Federal da Paraíba, João Pessoa, PB, Brasil; 6 Faculdade de Ciências Médicas, Departamento de Medicina, João Pessoa, PB, Brasil; 7 Departamento de Análises Clínicas, Toxicologia e Ciências dos Alimentos, Faculdade de Ciências Farmacêuticas de Ribeirão Preto, Universidade de São Paulo, Ribeirão Preto, SP, Brasil; 8 Department of Biomedical Genetics, University of Rochester, NY, USA; 9 Programa de Pós-graduação em Medicina e Saúde, Faculdade de Medicina, Universidade Federal da Bahia, Salvador, BA, Brasil

**Keywords:** Selenium, COVID-19, thyroid, nonthyroidal illness syndrome

## Abstract

**Objective:**

This study aimed to assess urinary selenium concentration (USC) and its correlation with non-thyroidal illness syndrome (NTIS) and inflammatory markers in hospitalized adult patients with COVID-19.

**Subjects and methods:**

A prospective study was conducted to investigate urinary selenium (Se) concentration in adult patients hospitalized with COVID-19 between June and August 2020. Urine and serum samples were collected before complications occurred, always within the first 48 hours after onset. A total of 121 patients were stratified into three tertiles based on USC: (i) USC < 25 µg/L (^40^), (ii) USC 25-39 µg/L (^41^), and (iii) USC > 39 µg/L (^40^). ICP-MS was employed to measure urinary Se concentration. NTIS was defined by free triiodothyronine below 2.3 pg/L accompanied by low or normal thyroid-stimulating hormone levels.

**Results:**

NTIS was observed in a low prevalence (5.7%) and was significantly associated with patients having the lowest USC (n = 6, p = 0.008). Thyroiditis was the most prevalent thyroid complication (23.9%); however, there was no significant association with USC (p > 0.05).

**Conclusion:**

The association between low USC and NTIS was evident in this cohort.

## INTRODUCTION

Selenium (Se) is an essential trace element for the formation of selenoenzymes, crucial for the proper functioning of the immune, antioxidant, reproductive, and thyroid hormone systems (^1^). Enzymes such as glutathione peroxidases (GPx), thioredoxin reductases (TRx), and iodothyronine deiodinases play vital roles in cellular redox control and thyroid hormone biosynthesis (^2^).

Individuals deficient in Se are more susceptible to impaired immune function, thyroid autoimmune disease, cognitive decline, and increased viral virulence, among other conditions (^3^). Since the optimal Se level depends on various factors, the literature suggests that an adequate serum Se level ranges between 90-120 µg/L, while the recommended daily intake of this micronutrient should be between 40-400 µg/day (^4^,^5^).

In the context of COVID-19, Se deficiency has been linked to increased oxidative damage caused by the SARS-CoV-2 virus, leading to a higher risk of severe disease and mortality (^6^-^10^). The elevated levels of reactive oxygen species in patients with COVID-19, coupled with lower levels of the selenoproteins GPxs and TRx, contribute to increased virulence (^10^,^11^). Furthermore, scientific findings have shown that low Se levels are associated with altered T cell differentiation, macrophage function, and synthesis of interferon-gamma and tumor necrosis factor-alpha (^11^,^12^).

Studies have shown a bidirectional relationship between COVID-19 and the occurrence of thyroid disorders such as thyroiditis, hypothyroidism, and an increased prevalence of NTIS (^13^-^15^). NTIS, frequently observed in critical illnesses, is characterized by changes in thyroid hormone (TH) metabolism unrelated to functional abnormalities in the hypothalamic-pituitary-thyroid axis (^16^). Noteworthy hormonal changes in this condition include reduced triiodothyronine (T3), increased reverse triiodothyronine (rT3), normal or slightly decreased thyroid stimulating hormone (TSH), and elevated concentrations of interleukin 6 (IL-6) (^17^). Elevated inflammatory cytokines, associated with COVID-19 and NTIS (^14^,^15^,^18^), suggest that Se deficiency may contribute to the onset of NTIS through its antioxidant properties and role in thyroid hormone regulation (^1^).

The potential risk of Se as a factor for diabetes, insulin resistance, hypertension, and other COVID-19 comorbidities has been explored for several years within the scientific community, yielding differing opinions. However, limited studies have examined the association between Se and NTIS in hospitalized patients with COVID-19. Our previous research focused on clinical, hormonal, and genetic aspects related to COVID-19 mortality in Brazil (^19^). Nonetheless, the nutritional status of Se in our population has remained poorly understood. Therefore, this study aims to investigate the nutritional status of Se and its association with NTIS in non-critical hospitalized patients with COVID-19.

## SUBJECTS AND METHODS

### Subjects and data collection

This cross-sectional study was aligned with another prospective, observational, analytical study conducted between June and August 2020. A total of 121 consecutive patients with confirmed COVID-19 were recruited from Hospital Metropolitano Dom José Maria Pires, a tertiary referral hospital in João Pessoa, Paraíba, Brazil. Written consent was obtained from the patients’ legal representatives, and the study received approval from the Ethics Committee in Research with Human Beings of the University Hospital Lauro Wanderley (CAAE no. 43097115.2.0000.5188). The study was conducted in accordance with the Declaration of Helsinki and complied with local and national regulations.

All patients tested positive for SARS-CoV-2 using real-time quantitative reverse transcriptase-polymerase chain reaction (Biomol OneStep/COVID-19, IBMP, Paraná, Brazil) with respiratory tract samples (^20^). Detailed information regarding patient selection has been previously published (^19^).

### Procedures

Patients over 18 years of age with COVID-19 who were admitted to the hospital’s nursing units were included in this research. Blood and urine samples were collected within the first 48 hours of hospitalization, before administering any interventions or therapies that could potentially interfere with or alter the results of Se, TH, or cytokine serum levels.

### Urinary selenium concentrations

Standard operational procedures were followed for collecting, storing, and transporting urine samples. The University of São Paulo’s Laboratory of Toxicology and Essentiality of Metals in Ribeirão Preto, Brazil, performed the USC measurements using inductively coupled plasma mass spectrometry (ICP-MS) (PerkinElmer, NexION^®^ 2000, Waltham, USA).

For the USC analysis, a 1:20 dilution was prepared in 0.5% nitric acid and 0.005% Triton. An analytical curve was constructed using the multielement standard solution (PerkinElmer), with concentrations ranging from 0.5 to 100 ppb and base urine for matrix-matched calibration curves. Selenium was the monitored isotope, and all materials used were previously decontaminated with 10% nitric acid for 24 hours (^21^).

The principle of the analytical technique, ICP-MS, involves using a plasma source energized through electrical currents generated by electromagnetic induction achieved by varying magnetic fields over time. This technique enables multielement analysis of traces, typically at parts per trillion levels (^21^). Notably, ICP-MS possesses a high detection capacity, capable of detecting 20-30 metals within minutes, even at significantly low concentrations. Specifically for Se, the urinary detection limit is 0.104 µg/L (^22^). Analyses were performed in triplicate, and the results were expressed in µg/L.

Given the absence of a universal classification for USC in hospitalized patients, this study considered the reference values for a healthy adult population (10-110 µg/L) (^23^).

### Serum biochemistry and NTIS diagnosis

C-reactive protein (CRP), D-dimer, lactate dehydrogenase (LDH), and IL-6 were measured using a chemiluminescence immunoassay (Maglumi-2000-Plus, Shenzhen New Industries Biomedical Engineering Co., Shenzhen, China) in accordance with the manufacturer’s protocol. According to the 2013 consensus of the Brazilian Society of Endocrinology and Metabolism, hypothyroidism is characterized by a TSH level above 5 µUI/mL and free thyroxine (fT_4_) below 0.89 ng/dL, hyperthyroidism by TSH below 0.4 µUI/mL and fT_4_ above 1.72 ng/dL, and NTIS by a serum free triiodothyronine (fT_3_) below 2.3 pg/L, accompanied by low or normal TSH levels (^24^).

### Statistical analysis

The data, including levels of IL-6, LDH, CRP, D-dimer, age, and body mass index (BMI), were expressed as the median ± interquartile range. Statistical comparisons between different Se tertiles and other groups were conducted using the Mann-Whitney test for continuous variables and the Cochran-Armitage test for categorical variables. The Kruskal-Wallis test was applied to compare all biochemical variables across the tertiles.

Given the lack of reference parameters for urinary Se in individuals with health conditions, the population sample was divided into tertiles. The p-value was employed to reject the null hypothesis and to ascertain the likelihood of differences between groups or variables.

## RESULTS

The mean total USC was 33.8 (standard deviation = 18.8 µg/L) with a median of 31.0 µg/L. Among the patients studied, 114 (94.2%) had USC levels within the normal range for healthy individuals (10-110 µg/L). The minimum USC observed was 5 µg/L, while the maximum reached 142.9 µg/L. Only seven patients exhibited USC levels below 10 µg/L, represen­ting 5.8% of the study population. **^Table 1^** demonstrates the association between urinary Se concentration and sociodemographic as well as clinical variables in the study population. The 121 patients were categorized into three groups based on USC tertiles: (i) USC < 25 µg/L (n = 40), (ii) 25 < USC < 39 µg/L (n = 41), and (iii) USC > 39 µg/L (n = 40) (**^Table 1^**). A significant number of patients in the lowest USC tertile were elderly (n = 22; 55%) (*p* < 0.05). With respect to BMI, a majority of obese individuals were found in the highest USC tertile (n = 28; 78.6%) (*p* < 0.05).

**Table 1:** Demographic and clinical characteristics of the patient cohort and their association with thyroid complications and urinary selenium concentration

Variables	Mann-Whitney and Cochran-Armitage tests				
	Total (n = 121)	USC < 25 (n = 40)	25 < USC < 39 (n = 41)	USC > 39 (n = 40)	p
Median age (IQR)	62 (48-75)	66 (55-77)	57 (48-76.5)	56.5 (45-67)	0.047*
BMI (kg/m^2^)	30.5 (27-34)	29.7 (27-32)	29.1 (24-32)	32.6 (29-39)	0.001*
Age > 60 years (%)	52 (42.9)	22 (55)	17 (41.4)	13 (32.5)	0.004*
Male (%)	75 (61.9)	25 (62.5)	27 (65.8)	23 (57.5)	0.212
**Thyroid complications**					
NTIS (%)	7 (5.7)	6 (15)	1 (2.4)	0 (0)	0.008*
Thyroiditis (%)	29 (23.9)	9 (22.5)	12 (29.2)	8 (20)	0.06

The Mann-Whitney test was utilized for continuous variables, whereas the Cochran-Armitage test was applied to all other variables. BMI: body mass index; IQR: interquartile range; NTIS: nonthyroidal illness syndrome; USC: urinary selenium concentration. *p < 0.05" statistically significant.

Thyroiditis emerged as the most prevalent thyroid complication, accounting for 23.9% of the cases, yet it did not show a significant association with USC levels (*p* > 0.05). Conversely, NTIS, despite its relatively low prevalence (5.75%), was associated with lower USC levels (*p* < 0.05) (**^Table 1^**).

## DISCUSSION

Previous findings within this cohort have demonstrated a clear association between serum thyroid hormone levels, inflammatory response, disease severity, and lethality (^19^,^25^). However, existing data directly correlating the interplay between Se, COVID-19, and NTIS are limited, underscoring the novelty and importance of the present study in elucidating this relationship.

COVID-19 is characterized by a heightened inflammatory state due to dysregulated cytokine production (^26^). This elevation in inflammatory cytokines overwhelms the immune system, leading to complications such as multiple organ dysfunctions, increased mortality risk, and organic sequelae (^27^). Numerous studies have corroborated the inflammatory profile of COVID-19, with serum levels of pro-inflammatory biomarkers, including LDH, CRP, D-dimer, and IL-6, being significantly elevated upon admission in both critical and non-critical patients across multiple clinical trials (^28^-^30^).

Excessive cytokine production may contribute to the development of NTIS in the studied population, as pro-inflammatory proteins are associated with its occurrence in critically ill hospitalized patients (^29^,^31^,^32^). It is pertinent to note that Se deficiency is associated with the occurrence and progression of viral and bacterial infections, including human immunodeficiency virus, poliovirus, and tuberculosis (^11^). Thus, the study population is estimated to be susceptible to the same scenario.

The heightened inflammatory state indicates intense defense system activation, potentially affecting Se availability and depleting its reserves. The hyperresponsiveness to inflammation and immunological demand in COVID-19 underscores the importance of antioxidants and modulators in inflammation control, highlighting Se’s critical role at this stage (^18^). Adequate Se levels in this population could beneficially impact the balance between self-reactive disease activity and thyroid function, given Se’s essential contributions to this system (^33^). Hence, it is suggested that lower USC favored the imbalance between the reduction in amount or activity of thyroid selenoproteins, such as glutathione peroxidase type 3 and selenoprotein S, and the pronounced inflammatory state in the population (^29^,^31^,^32^).

Several studies have demonstrated the correlation between pro-inflammatory cytokines, specifically interleukin 1β, IL-6, and tumor necrosis factor-alpha, and the onset of metabolic alterations in TH and NTIS. In the context of critical conditions and highly inflammatory diseases such as COVID-19, there is an alteration in the metabolism of TH through the induction of iodothyronine deiodinase 3, a Se-dependent enzyme (^34^-^36^). This alteration leads to an increase in T_3_ clearance and rT_3_ production. Additionally, critical illness also results in a decreased expression of iodothyronine deiodinase 1 (D1) in the liver, consequently reducing the production and clearance of T_3_r (^36^). This phenomenon appears to be linked with increased free radical generation in tissues, which can decrease1 activity due to oxidative damage to this enzyme (^37^). Furthermore, elevated circulating glucocorticoids, a drug class recommended in severe COVID-19 cases, may also diminish D1 activity (^38^).

In an *in vitro* study, sodium selenite was shown to partially counteract the oxidative stress generated by high concentrations of IL-6 induced in a cellular model simulating NTIS, thereby providing evidence of Se’s role in modulating the disease’s underlying mechanisms (^39^). Another randomized controlled trial involving 68 dialysis patients who received high-dose Se supplementation (200 µg/day for 12 weeks) demonstrated a reduction in rT_3_ levels (^40^). These studies, along with the findings from the present cohort, underscore the significant contribution of Se to thyroid health, particularly in patients with COVID-19 (^2^).

In our cohort, we previously reported a statistically significant association between serum fT_3_ (a marker of NTIS), CRP, neutrophil count, serum IL-6, albumin, and the D-dimer ratio with mortality (^25^). Additionally, in assessing the influence of BMI on the USC of our cohort, we found that patients with higher USC exhibited a greater BMI, which was significantly associated when patients were stratified into USC tertiles. This observation requires further confirmation in future studies, especially due to the altered micronutrient intake prior to hospital admission caused by the severity of the disease.

USC offered more feasibility in our study, given that 50%-70% of dietary Se is excreted through urine, and a single urine collection is practical and less invasive. It has been utilized in epidemiological studies such as the National Health and Nutrition Examination Survey III and the Canadian Health Measures Survey, as well as in biomonitoring studies assessing metal exposure. Conversely, serum Se assessment is invasive, requires trained professionals for analysis, and is more time-consuming (^41^,^43^).

Some limitations of the current study include the non-association of Se status with certain data, such as the method’s specificity for a highly specific population, the lack of urinary Se reference parameters in ill individuals, and the potential interference from confounding factors. Including plasma markers, such as serum Se or plasma selenoprotein P could have mitigated the study’s multiple biases. Although we did not evaluate the activity of Se-dependent enzymes, it is established that GPx is essential for protecting the thyroid gland from oxidative damage and for regulating the availability of Se for selenoprotein synthesis, including deiodinases.

In the context of NTIS associated with COVID-19, a decrease in GPx activity due to Se deficiency, could correlate with diminished deiodinase activity and altered thyroid hormone metabolism, contributing to oxidative stress and impaired conversion of T_4_ to the active form T_3_. The reduction in these enzymes may further exacerbate the hormonal imbalance observed in hypothyroidism (^44^,^45^). However, practical challenges such as obtaining adequate blood sample aliquots for storage, transport, and analysis have made urinary samples a more cost-effective and feasible option in this context.

In conclusion, nonthyroidal illness syndrome emerged as the most significant complication in this study and was associated with low levels of urinary selenium in the affected population, likely due to elevated pro-inflammatory markers. This finding supports the importance of evaluating the selenium nutritional profile and its potential protective effects in hospitalized patients with COVID-19.

## Data Availability

the datasets generated and/or analyzed during the present study are available in the Institutional Repository of the Federal University of Paraíba at the link https://repositorio.ufpb.br/jspui/simpleearch?query=AVALIA%C3%87%C3%83O+DA+FUN%C3%87%C3%83O+TIREOIDIANA+E+POLIMORFISMO+THR92ALA-D2+DO+GENE+DA+DESIODASE+TIPO+2+COMO+BIOMARCADORES+DE+MORTALIDADE+NA+COVID-19++
